# Comprehensive Study of Total Nitrogen Content and Microfluidic Profiles in Additive-Enriched Plant-Based Drinks

**DOI:** 10.3390/foods13152329

**Published:** 2024-07-24

**Authors:** Fruzsina Balogh-Hartmann, Csilla Páger, Anita Bufa, Zoltán Sipos, Anna Dávidovics, Zsófia Verzár, Tamás Marosvölgyi, Lilla Makszin

**Affiliations:** 1 Institute of Bioanalysis, Medical School, Szentágothai Research Center, University of Pécs, 7622 Pécs, Hungary; fruzsina.hartmann@aok.pte.hu (F.B.-H.); csilla.pager@aok.pte.hu (C.P.); anita.bufa@aok.pte.hu (A.B.); sizfab.t.jpte@pte.hu (Z.S.); marosvolgyi.tamas@pte.hu (T.M.); 2 Department of Languages for Biomedical Purposes and Communication, Medical School, University of Pécs, 7624 Pécs, Hungary; anna.davidovics@aok.pte.hu; 3 Institute of Nutritional Sciences and Dietetics, Faculty of Health Sciences, University of Pécs, 7621 Pécs, Hungary; verzar.zsofia@pte.hu

**Keywords:** plant-based milk alternatives, kidney disease, microfluidic profile, total nitrogen content, microchip gel electrophoresis

## Abstract

The growing consumption of plant-based milk substitutes raises important questions about their composition. The various additives used by manufacturers, including those employed as flavor enhancers, protein additives, and stabilizers, may contain both protein and non-protein nitrogen components. In our study, we examined not only popular milk alternatives but also other milk substitutes made from specific plants. We present a reproducible and rapid method for the simultaneous qualitative and quantitative determination of the total nitrogen content in milk alternatives, focusing on applicability. Using the microchip gel electrophoretic method, we determined that the total nitrogen content differed from the protein content indicated on the packaging. Our results, along with statistical evaluations, supported the hypothesis that different brands of products, derived from the same plant source, resulted in different microfluidic profiles, likely due to the presence of additives. As expected, the microfluidic profiles of additive-free products differed from those of fortified products made from the same plant-based milk replacer. Total nitrogen content provides crucial information for individuals with kidney disease, as is essential to reduce the burden on the kidneys to slow deterioration, alleviate symptoms and avoid complications.

## 1. Introduction

One of the topical issues of the 21st century in a consumer society is the proper selection and intake of food from a wide range of choices. Around the world, it is important for people to eat consciously and to develop a proper diet [1,2,3,4,5,6]. This becomes even more critical for individuals with health problems as most people in such situations will carefully examine the ingredients listed on the packaging.

Plant-based milk replacers can be a solution for people with milk protein allergies and lactose intolerance, among other conditions [7]. In addition to other benefits, their high fiber, antioxidant and unsaturated fatty acid content can aid in preventing various diseases (e.g., cardiovascular disease) [3,8,9]. Protein intake is a relevant issue for patients with kidney disease [10,11]. Proteins are broken down into amino acids during digestion and the body uses these amino acids for different functions. However, during the metabolism of proteins, via urea, ammonia, creatinine and uric acid are produced as by-products. Healthy kidneys, with a GFR (glomerular filtration rate) of 100–120 mL/min, are usually efficient at filtering nitrogen-containing compounds, but in kidney disease, this process can be impaired, leading to a build-up of nitrogenous waste in the blood. There are five stages of chronic kidney disease based on GFR [11]. In both mild and advanced stages of chronic kidney disease, it is important to maintain a proper diet, including reducing protein consumption, to lessen the burden on the kidneys [10,11,12]. Excessive nitrogen accumulation can contribute to uremia, a condition where waste products build up in the bloodstream. This can lead to various complications, including fatigue, nausea, and, in severe cases, damage to other organs [10]. In the context of kidney disease, monitoring nitrogen intake is crucial, as nitrogen is a component of various compounds, including proteins and additives, which can contribute to the overall nitrogen load of the body [10,11].

While food labels generally provide information on protein content, nitrogen content is not necessarily indicated [13]. In the European Union, there is no requirement for the quantitative labeling of nitrogen content, as the importance of this nitrogenous compound is not yet widely recognized by the public. Proposals for quantitative labeling are being put forward by the European Commission. Nevertheless, the proposals for including nitrogen content in the labeling put forward by the European Commission represent a progressive step towards more comprehensive nutritional transparency. The inclusion of nitrogen content on food labels has the potential to enhance consumers’ understanding of the nutritional composition of their food, thereby encouraging more informed dietary decisions. As the importance of detailed nutritional information grows, this move could align with broader public health objectives and foster greater consumer awareness.

Compared to animal milks, most milk analogues lack the expected taste, texture, proteins, amino acids, and vitamin D content [8,14,15]. Plant-based milk alternatives (PBMAs) can be developed using various ingredients that can contribute to the nitrogen content, either in the form of proteins or other nitrogen-containing compounds [14,16]. Non-protein nitrogen (NPN) components can include the following compounds: free amino acids, nucleic acids, or other N-compounds [16]. However, the specific nitrogen additives may vary depending on the brand and composition of the product. Here are some common ingredients found in plant-based beverages that may contribute to the nitrogen content:

*Protein additives of plant origin* include pea protein, soy protein, coconut protein, rice protein, marine algae, red algae, chicory, chicory root fiber, sunflower and safflower oils, soluble corn starch, grape juice concentrate including grape seed, carob meal, and rosemary extract. These proteins contain nitrogen as part of their amino acid structure [16,17,18,19,20,21,22,23,24].

Food product developments favor alternative protein sources, including algae [18,20]. Marine algae have a high protein content (7–47 g/100 g dry weight) and adequate amino acid content, which is influenced by climatic factors. Additionally, they are rich in vitamins (vitamins B, C, A, and E) [18,21]. Vitamin B present in marine algae can also contribute significantly to nitrogen content [16].

*Emulsifiers, stabilizers, and foaming agents* such as lecithin [25,26], which is often used as an emulsifier and antioxidant, and stabilizers such as guar gum or xanthan gum contain nitrogen. They are used to improve the consistency and stability of the beverage, contributing mainly as NPN components to the total nitrogen concentration [15]. Some of the ingredients listed on the packaging include maltodextrins, which act as a foaming agent in the processing of the products. However, maltodextrins are also produced with sodium glutamate, which is considered an NPN ingredient, too.

Food technology uses *sweeteners*, such as certain sugar alcohols, artificial sweeteners, or flavorings, to impart the right taste to plant-based milk substitutes. These components may also contain nitrogen [16].

*Vitamins* (mainly vitamins B) added as additives to milk alternatives during food processing may contribute to NPN [16].

It is important to note that although these ingredients contribute to the overall nitrogen levels, they are not necessarily harmful to individuals with healthy kidneys. However, for people with kidney disease, managing the overall dietary nitrogen load is key.

This study reports on additive-containing milk alternatives made from various plants available on retail shelves in Hungary.

Our aims are to (*i*) determine the total nitrogen content (protein and non-protein nitrogen) of plant-based drinks and compare the protein content stated on the food labels with the measured nitrogen content; (*ii*) compare the microfluidic profile and total nitrogen content among products of the same type but from different brands and additives; (*iii*) compare the total nitrogen content and the microfluidic profile of the same type plant-based drink alternatives between additive-free and additive-enriched products; (*iv*) compare the electrophoretic profiles of PBMA from different origins to identify characteristic features. To the best of our knowledge, there are no prior studies that have performed an analysis of the total nitrogen content of PBMA using MGE.

## 2. Materials and Methods

### 2.1. Plant-Based Milk Alternative Samples

PBMAs purchased from local supermarkets in Hungary, a member state of the European Union, over the period 2019–2023 were selected for study. These PBMAs were packaged in cartons that required no refrigeration prior to opening and included a range of additives such as sugars, flavorings, emulsifiers, oils, and other plant-based components. A total of eighteen different PBMA varieties were tested, including seven soybeans, eleven coconuts, twenty types of rice, thirteen almonds, sixteen oats, four spelts, three wholegrain oats, two types of brown rice, one cashew, one hazelnut, one hemp, one quinoa, one Brazil nut, one buckwheat rice, one millet, one oat–hemp–flaxseed, one pea, and one wholegrain spelt. The soy, coconut, rice, brown rice, oat, wholegrain oat, spelt, and almond-based drinks were made by different brands, and a total of eighty-six drinks were measured. Following randomization, which was conducted prior to unpacking the PBMAs, the samples were incubated at 37 °C in a JULABO U3 circulating water bath (Julabo GmbH, Seelbach, Germany). They were stirred in their original packaging and, after homogenization, transferred to 10 mL centrifuge tubes and stored at −20 °C until analysis. For each product, duplicate analyses were performed on samples with three different expiry dates from each brand.

### 2.2. Microchip Gel Electrophoresis

High Sensitivity Protein 250 (HSP 250, Agilent Technologies, Santa Clara, CA, USA) LabChip kits and the Agilent 2100 Bioanalyzer with a laser-induced fluorescence (LIF) detector were used for protein size separation, as described previously [27]. This optimized method used in the study of additive-free PBMAs was also applied to additive-containing milk replacers for greater efficiency and an exact comparison. To briefly summarize the sample preparation procedure, 0.5 µL of 10-fold diluted dye was added to a 5 µL sample volume and incubated for 10 min at room temperature. Ethanolamine was added to the fluorescent dye-labeled samples (to bind excess dye, resulting in a system peak), followed by incubation as previously described. Samples were diluted with distilled water two or five times according to the nominal protein content indicated on the milk alternate packaging, aiming for a concentration up to 0.6 g/100 mL. To denature the proteins, 4 µL of the diluted sample was mixed with 2 µL of denaturing solution and incubated at 100 °C for 5 min and then centrifuged. The channels of the microchips were filled hydrodynamically with the gel matrix, and then the destaining solution and the ladder were filled in the appropriate place. The 6 µL volumes of the samples were applied to the numbered wells in the channels containing the PDMA-based linear separation gel. Samples were injected at 1000 V for 80 s (injection volume approximately 40 pL) and separation towards the anode was performed at 30 °C for 60 s at 1000 V. Each sample was measured in duplicate. Agilent 2100 Expert software was used to evaluate the results. For the qualitative and quantitative analysis of protein components, electropherograms were integrated manually and molecular weights were determined using calibration curves [28]. The time-corrected area under the curve (TCA) of each protein fraction was determined and expressed as a percentage of their presence. Since the fluorescent dye covalently binds to free amino groups and forms a complex, it is possible to determine not only the protein content but also the total nitrogen content.

### 2.3. Statistical Analysis

The percentage of time-corrected area values was subjected to analysis using the statistical software package SPSS, version 28.0 (IBM, New York, NY, USA). The initial step was to assess the normality of the data distribution, which was subsequently rejected. The variables are presented as median values with the interquartile range. The Wilcoxon signed-rank test was utilized to identify any differences between the total protein content per 100 mL and the measured total nitrogen content. Furthermore, the Kruskal–Wallis test was employed with multiple pairwise comparisons to evaluate differences in total nitrogen content among different brands of the same types of plant-based drinks. *p*-values less than 0.05 were considered statistically significant. To examine the variability in the nitrogen content of different plant-based drinks, we performed a principal component analysis (PCA) with varimax rotation in the R statistical environment (R version 4.3.2, R Core Team (2023), Vienna, Austria).

## 3. Results and Discussion

### 3.1. Total Nitrogen Content Comparison

Our previous work presented the protein analysis of PBMA which did not contain any additives with MGE [27]. The present study investigated a total of eighty-six plant-based drinks with different types such as soy, coconut, rice, almond, oat, hazelnut, cashew, spelt, wholegrain oat, brown rice, hemp, quinoa, Brazil nut, buckwheat rice, millet, oat–hemp–flaxseed, pea, and wholegrain spelt which contain protein and non-protein nitrogen (NPN) additives.

In accordance with the EU Council Regulation no. 1169/2011 [13], all plant-based drinks are required to provide nutritional information, containing the total protein content (g/100 mL). This article presents an analysis of the total nitrogen content (protein and non-protein nitrogen) of various PBMAs. Total nitrogen content was calculated in accordance with the original protocol [28]. In order to ascertain the total nitrogen content of plant-based drinks, the concentration and area below the time-corrected peak of the ladder were taken into account, in addition to the dilutions employed in the preparation of the samples.

The evaluation of total nitrogen (TN) concentrations compared to nominal total protein (TP) values is shown in Table 1A,B.

The total nitrogen content of the plant-based drinks that were subjected to analysis was as follows: 2.0 (1.1–6.4) g/100 mL of oat drink, 0.7 (0.2–1.2) g/100 mL of rice drink, 1.8 (1.2–4.4) g/100 mL of coconut drink, 3.7 (1.8–8.3) g/100 mL of soy drink, 8.8 (6.1–12.2) g/100 mL of almond drink, 8.5 (3.8–9.0) g/100 mL of wholegrain oat, and 1.5 (0.7–3.5) g/100 mL of spelt drink. The microchip gel electrophoretic HSP 250 method allows a tolerance of ±20% CV (coefficient of variation) for the reproducibility of the peak area for total nitrogen concentration determination. Statistical analysis showed that the measured total nitrogen content, when compared to the total protein content on the label, differed significantly for oat, rice, coconut, and almond (*p* < 0.001) but did not differ significantly for soybean (*p* = 0.227), wholegrain oat (*p* = 0.080), and spelt (*p* = 0.116). The results indicated that the measured total nitrogen content (measured TN) in these drinks may have exceeded the value stated on the label (nominal TP) (Table 1A) because of the protein and NPN additives.

The total nitrogen content of the plant-based drinks that were subjected to analysis was as follows: 5.0 (2.4–10.5) g/100 mL of the hazelnut drink, 5.2 (1.9–14.5) g/100 mL of the cashew drink, 2.8 (2.3–2.9) g/100 mL of the wholegrain spelt drink, 1.0 (0.7–1.5) g/100 mL of the quinoa drink, 8.3 (5.1–15.1) g/100 mL of the Brazil nut drink, 0.9 (0.3–2.1) g/100 mL of the brown rice drink, 0.8 (0.6–1.4) g/100 mL of the buckwheat rice drink, 0.9 (0.8–1.2) g/100 mL of millet, 8.6 (6.9–14.2) g/100 mL of the hemp drink, 6.7 (4.3–8.5) g/100 mL of the oat–flax–hemp drink, and 5.5 (2.1–8.1) g/100 mL of the pea drink. Statistical analysis showed that the measured total nitrogen content, when compared to the total protein content on the label, differed significantly for hazelnut (*p* = 0.018), cashew (*p* = 0.026), wholegrain spelt (*p* = 0.014), Brazil nut (*p* = 0.026), hemp (*p* = 0.012), and oat–flax–hemp (*p* = 0.028) but did not differ significantly for quinoa (*p* = 0.746), brown rice (*p* = 0.122), buckwheat rice (*p* = 0.916), millet (*p* = 0.812), and pea (*p* = 0.113). Due to the low sample size, the reliability of these statistical values is lower.

### 3.2. Plant-Based Milk Alternatives Enriched With Additives

**Figure 1:**
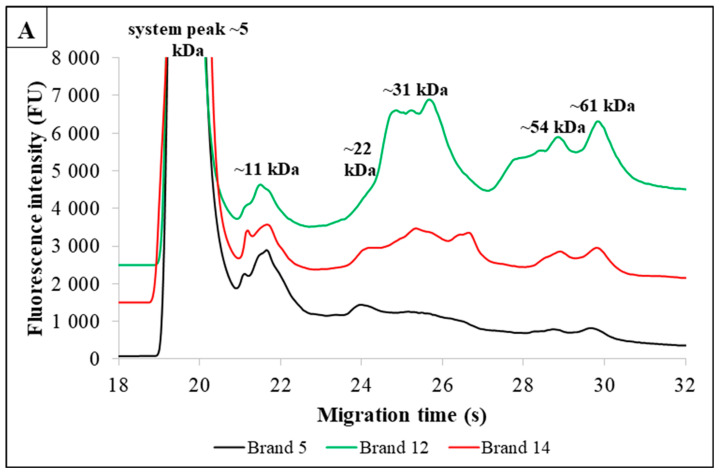

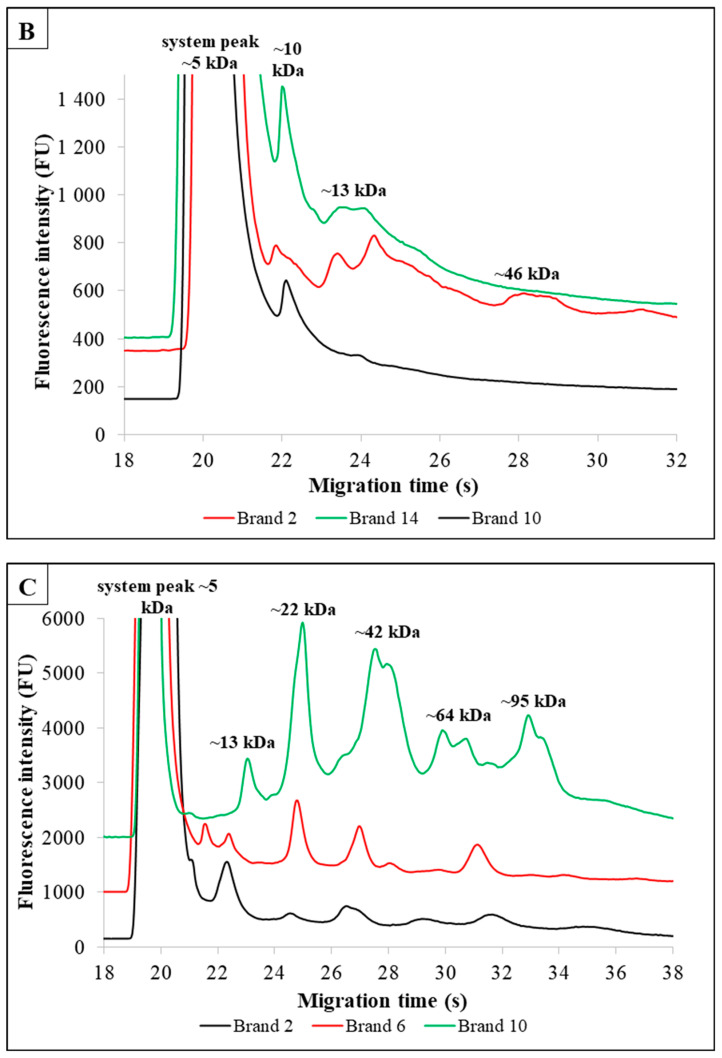

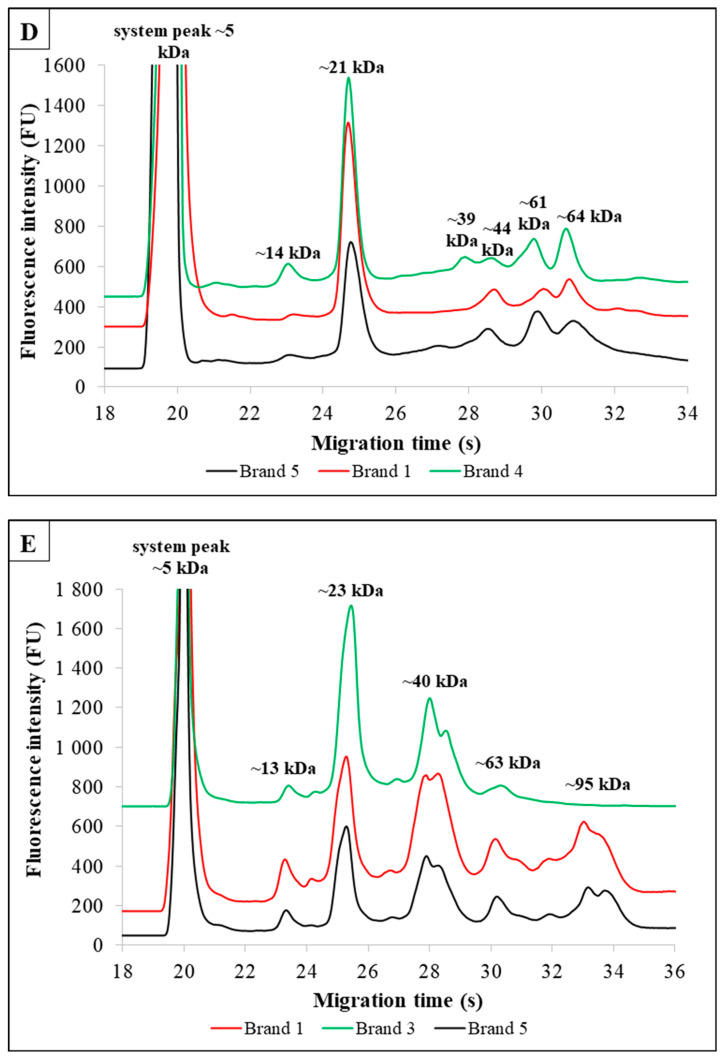

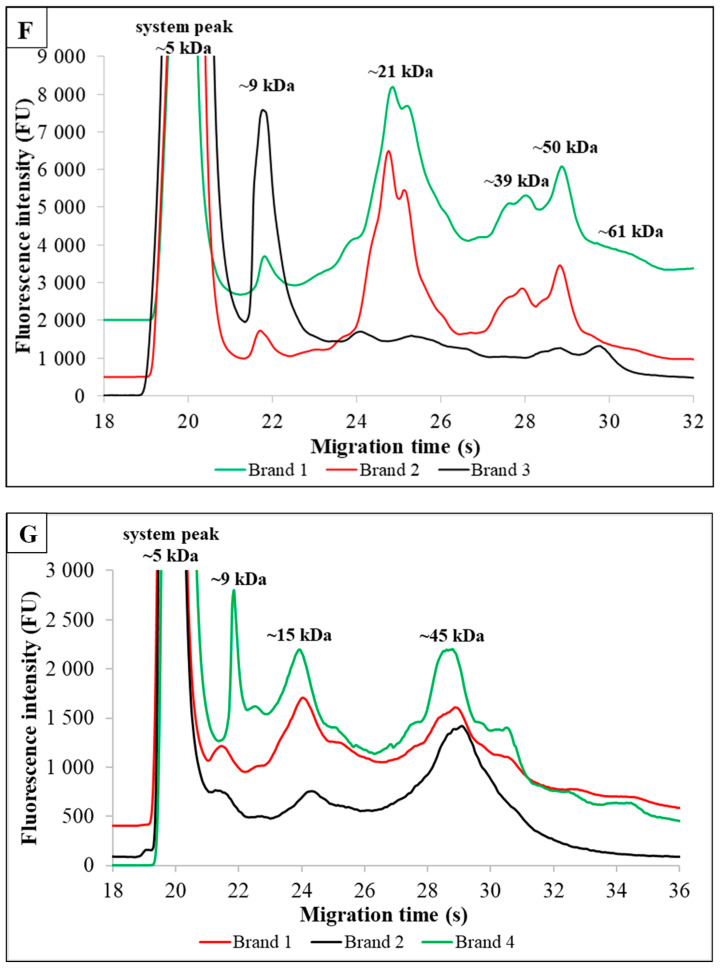
(**A**–**G**) Microfluidic profiles of seven plant-based drinks (three brands) with different additive contents. The Section 2.2 includes the optimized experimental method and a description of the conditions used. (**A**) illustrates oat-based drinks, (**B**) rice-based drinks, (**C**) coconut-based drinks, (**D**) almond-based drinks, (**E**) soybean-based drinks, (**F**) wholegrain oat-based drinks and (**G**) spelt-based drinks.

Figure 1 shows the electropherograms of three different brands of seven PBMAs, each containing various additives.

The addition of different additives to *oat-based* drinks from different brands results in unique profiles (Figure 1A) that exhibit similarities in certain aspects. The information obtained from the measurements showed that Brand 5 and Brand 12 resulted in four fractions, fraction 1: ~11 kDa, fraction 2: ~31 kDa, fraction 3: ~54 kDa and fraction 4: ~61 kDa, for MWs. In addition to these components, Brands 5 and 14 showed the presence of one more component at ~24 s migration time with a molecular weight of ~22 kDa. Brand 5 contained sunflower oil and marine red algae. Brand 14 contained safflower oil in addition to the additives found in Brand 5, suggesting that the difference between these two brands could be due to the presence of safflower oil. However, Brand 12 differs from the previous brands in that it contains pea protein as an ingredient. Despite the number of added components, it is the latter (Brand 12) that has the most pronounced fluorescence intensity peaks, which can be attributed to the presence of protein-rich peas [17]. A statistical evaluation of the TN content of the oat-based milk alternatives (16 different brands) showed a significant difference (*p* = 0.006), indicating that the different additives alter the total nitrogen content of these products to some extent.

*Rice-based* drinks also indicated several additives on the packaging. Three different additive contents, commonly used in rice drinks, were investigated. Brand 2 contained sunflower oil as an additive, Brand 10 contained sunflower oil, rapeseed oil, gellan gum and vitamins B2, B12, and D, and Brand 14 contained sunflower oil, safflower oil, and marine algae as additives. The three brands showed different profiles (Figure 1B). Four fractions were reported for Brand 2: fraction 1: ~9 kDa, fraction 2: ~12 kDa, fraction 3: ~15 kDa, fraction 4: ~46 kDa molecular weights. For Brand 10, a single fraction with a molecular weight of ~10 kDa appeared. Lastly, for Brand 14, two fractions with molecular weights of ~10 kDa and ~13 kDa appeared. The fractions were present in different numbers and intensities in each product but were visibly more intense in the marine algae sample (Brand 14) [18]. A significant difference (*p* = 0.039) was observed between the rice-based milk alternatives (20 different brands) based on the Kruskal–Wallis test.

The range of additives in *coconut* drinks is also diverse. Five-to-five protein fractions were determined for Brand 2, Brand 6, and Brand 10 (Figure 1C). The profile of Brand 2 shows the following components: fraction 1: ~11 kDa, fraction 2: ~17 kDa, fraction 3: ~40 kDa, fraction 4: ~60 kDa, and fraction 5: ~85 kDa with MW. For Brand 6, the fractions produced peaks with MWs at ~10 kDa, ~12 kDa, ~20 kDa, ~40 kDa, and ~75 kDa. When measured for Brand 10, fraction 1: ~13 kDa, fraction 2: ~22 kDa, fraction 3: ~42 kDa, fraction 4: ~64 kDa, and fraction 5: ~95 kDa appeared on the electropherogram. The packaging of Brand 2 showed that it contained grape juice concentrate, fatty acid sucrose esters, carotene, vitamins D2 and B12, the packaging of Brand 6 indicated rice, sunflower lecithin, gellan gum, guar gum, vitamin D, and B12, and the packaging of Brand 10 showed that it contained soybean. The main difference in Brand 6 compared to Brand 2 is due to the presence of sunflower lecithin. Furthermore, the electropherogram of Brand 10 is similar to the typical electropherogram of soybean (Figure 1E), due to the high protein content of soy [19,29]. Therefore, the Brand 10 profile is qualitatively and quantitatively different from the electropherograms of Brand 2 and Brand 6. No significant difference (*p* = 0.054) was found for this number of elements in the Kruskal–Wallis test for coconut drinks (11 different brands).

The packaging of *almond-based* drinks also displayed several additives. The objective was to investigate three different additive contents, which are commonly used in almond drinks. The additives present in Brand 4 were carob meal, maize maltodextrin, and gellan gum. In Brand 5, gellan gum and guar gum were identified. Finally, Brand 1 was found to contain sunflower lecithin, carob meal, gellan gum, and vitamin B. Six fractions were reported for Brand 4, with the following molecular weights: fraction 1: ~14 kDa, fraction 2: ~21 kDa, fraction 3: ~39 kDa, fraction 4: ~44 kDa, fraction 5: ~61 kDa and fraction 6: ~64 kDa. In the case of Brands 5 and 1, a single fraction with a molecular weight of ~39 kDa was absent, likely due to the absence of maize maltodextrin (Figure 1D). The fractions were present in varying numbers and intensities in each sample. A significant difference (*p* = 0.002) was observed between the almond-based milk alternatives (13 different brands) based on the Kruskal–Wallis test.

The most common additives in *soybean-based* drink alternatives are marine algae, various vitamins, and gellan gum. Brand 1 contained cane sugar and marine algae, Brand 3 contained gellan gum, vitamins B12, D, and B2, and Brand 5 contained calcium carbonate, gellan gum, vitamins B2, B12, and D2, and marine algae. The electrophoretic profiles (Figure 1E) showed 4 protein fractions (~13 kDa, 23 kDa, 40 kDa, 63 kDa) in the Brand 3 sample, and a fifth protein fraction peak with ~95 kDa MW appeared in the Brand 1 and Brand 5 samples, probably due to marine algae content. Kruskal–Wallis statistical analysis showed no significant difference (*p* = 0.741) between the soy-based drinks (seven different brands) in terms of total nitrogen (TN) content.

For the *wholegrain oat-based* milk alternatives, three different brands were presented (Figure 1F). Brand 1 contained sunflower oil, Brand 2 also contained sunflower oil, and Brand 3 contained sunflower oil and marine algae as added ingredients. In addition to the system peak, four peaks (~9 kDa, 21 kDa, 39 kDa, and 50 kDa) appeared in the electrophoretic profiles of Brand 1 and Brand 2. These components were similar not only qualitatively but also quantitatively. In the case of Brand 3, the most prominent peak was observed at ~9 kDa MW. It can thus be determined that, while samples containing similar additives displayed similar profiles, different wholegrain oat drinks (containing marine algae) showed different electropherograms. The statistical evaluation showed no significant difference between the TN concentrations of the whole grain oat drinks (three different brands) at this element count (*p* = 0.344).

For *spelt-based* drinks, three brands were tested, all containing only sunflower oil as an additive. The figure shows the electropherograms of these three brands (Figure 1G), where the similarity of the profiles can be observed. The differences in the fluorescence intensity of the fractions may be due to differences in the spelt content of the products. Three fractions (~9 kDa, 15 kDa, and 45 kDa) can be observed for each brand. In the statistical evaluation, no significant difference was determined for the total nitrogen content of spelt-based drinks (four different brands) using the Kruskal–Wallis test (*p* = 0.300).

In summary, based on the Kruskal–Wallis test, there was a significant difference in the total nitrogen content between brands of oat-based, rice-based, and almond-based plant drinks, but no significant difference was observed in the total nitrogen content between brands of coconut-based, soy-based, wholegrain oat-based, and spelt-based drinks.

### 3.3. Comparison of Non-Additive and Additive-Enriched Products of the Same Type of Plant-Based Milk Alternatives

**Figure 2:**
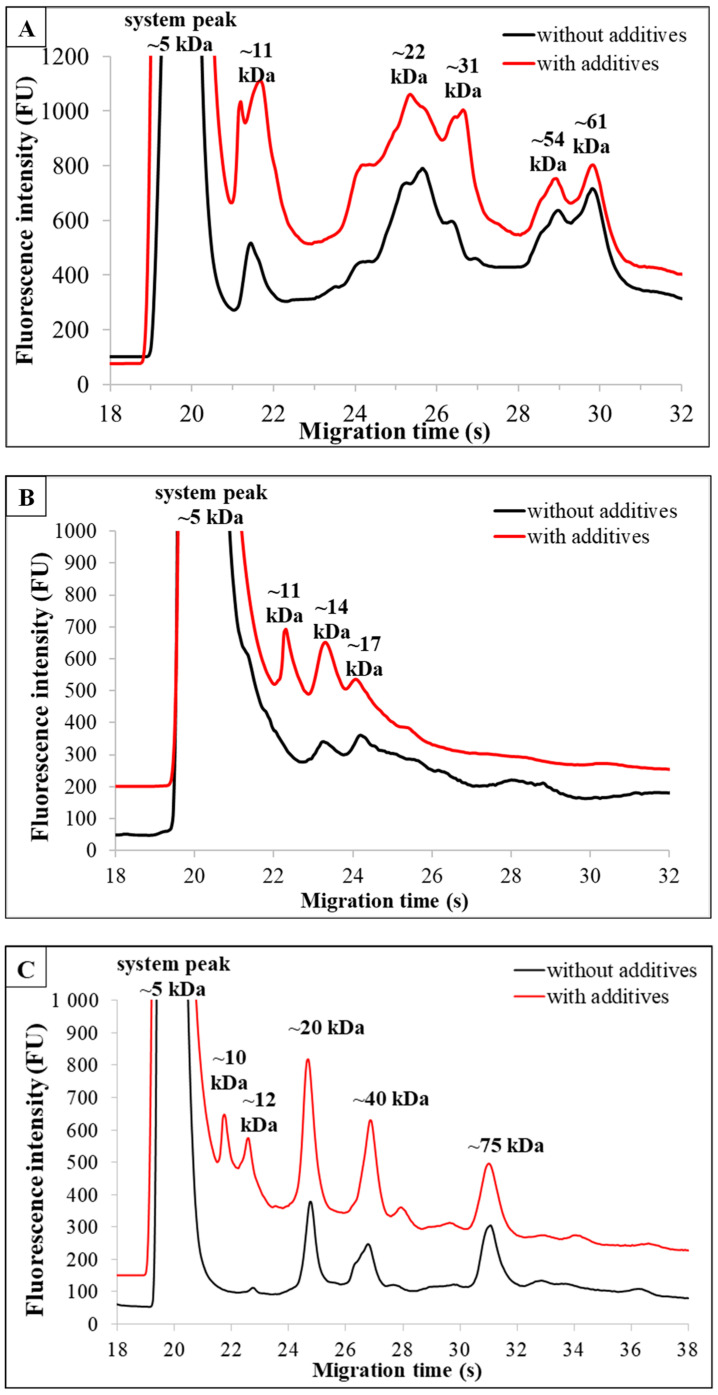

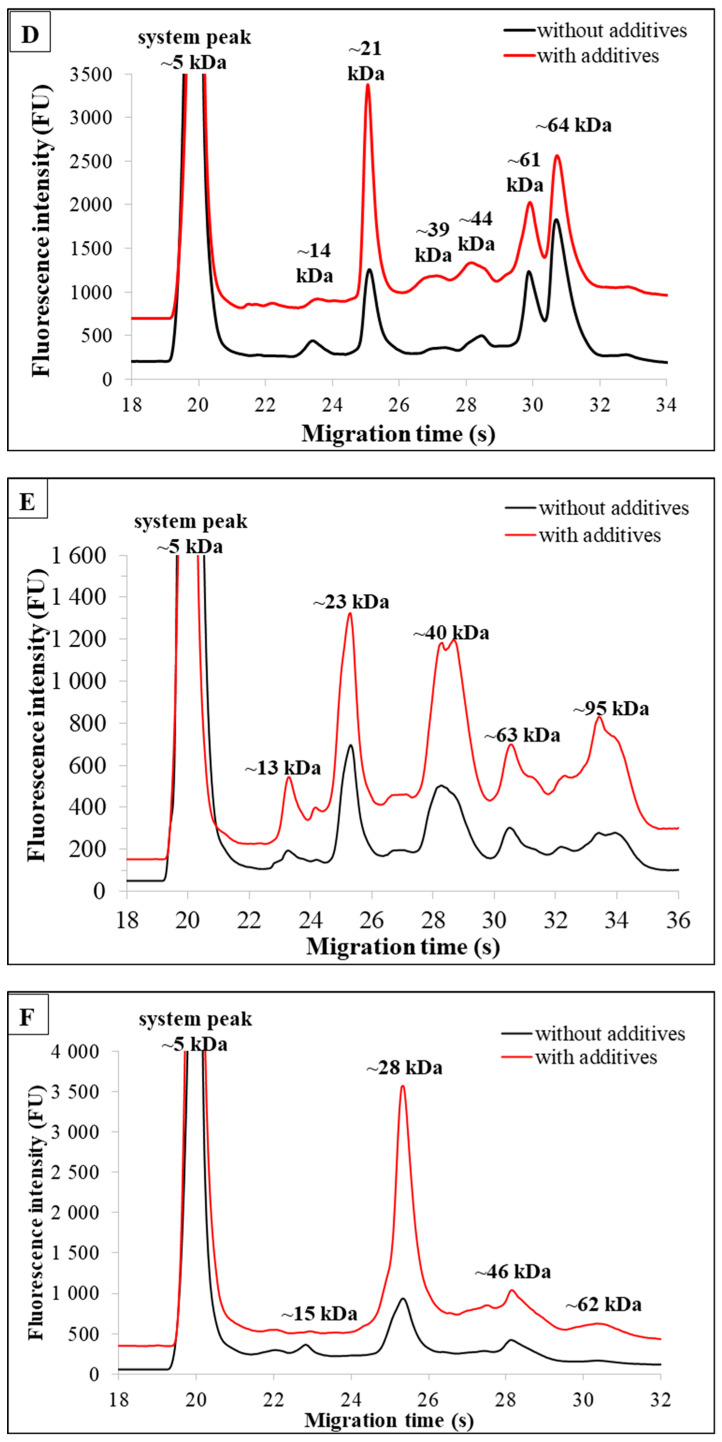
(**A**–**F**) The figures show a comparison of the microfluidic profiles of different plant-based milk replacers without additives (black line) and additive-enriched (red line). (**A**) illustrates oat-based drinks, (**B**) rice-based drinks, (**C**) coconut-based drinks, (**D**) almond-based drinks, (**E**) soybean-based drinks, and (**F**) cashew-based drinks.

In order to gain insight into the impact of additives on PBMAs’ microfluidic profiles, we selected brands for comparison whose additives exhibited the most significant influence. The electropherograms (Figure 2A–F) illustrate the differences between milk alternatives with (red line) or without (black line) additives [27]. In some cases, there were differences observed both qualitatively and quantitatively. Different additives had various effects on the electropherograms.

For *oat-based* drinks, a milk replacer containing 14% oats, sunflower oil, safflower oil, marine algae, and salt resulted in a distinct profile (Figure 2A) compared to the one without additives due to the added excipients. Variations in the ratio of fractions and new components were observed in the product with additives.

Samples of *rice-based* drinks were compared where only sunflower oil was indicated as an additive on the product label. Figure 2B illustrates that despite only one indicated difference (~11 kDa) in ingredients between the two products, several fractions appeared with higher intensity on the electropherogram for the fortified product.

The *coconut milk-based* drinks were selected to include rice, vitamin B12, and texture enhancers such as gellan gum, guar gum, and sunflower lecithin, in addition to coconut. The electropherograms (Figure 2C) illustrate the difference between the two products. The additive-enriched sample produced five main and three minor peaks, but the additive-free product produced five components, hence the difference in the number of components in the milk drinks. The difference is due to the presence of additives.

In the case of *almond-based* drinks, the electropherograms of both additive-enriched and additive-free products (Figure 2D) revealed six fractions for the milk alternatives made solely from almonds, salt, and water, as well as for the product containing additives. These fractions were either qualitatively or quantitatively similar. The sample with additives listed guar gum, gellan gum, and lecithins on the packaging.

The electropherograms obtained from microchip gel electrophoresis measurements showed 5–5 protein fractions for both *soy-based* drinks (Figure 2E). The peaks of the components determined in the additive-free and additive-containing samples showed qualitative agreement. The fractions consistently appeared in the profile with the following molecular weights for both products: ~13 kDa, ~23 kDa, ~40 kDa, ~63 kDa, and ~95 kDa MW [27]. Furthermore, the abundances of the components of the additive-enriched product were similar to those of the additive-free product [27]. The intensities of the fractions are visibly different, with the additive-enriched product producing higher intensity peaks, mainly due to the marine algae content.

For *cashew-based* drinks with additives, the electrophoretic profile (Figure 2F) could have been influenced by several additional components, including carob meal, gellan gum, sunflower lecithin, and various vitamins (B2, B12, E, and D2). The peaks generated by the protein and NPN components did not differ in number or quality from those identified in the additive-free sample. However, when comparing the intensities of the individual components, fraction 2 (~25 s, ~28 kDa MW) was notably more prominent in the enriched sample than in the additive-free sample.

Overall, it can be concluded that the protein components and NPN components used as additives contribute to the electrophoretic profile and total nitrogen content.

### 3.4. Special Plant-Based Milk Alternatives Enriched With Additives

**Figure 3:**
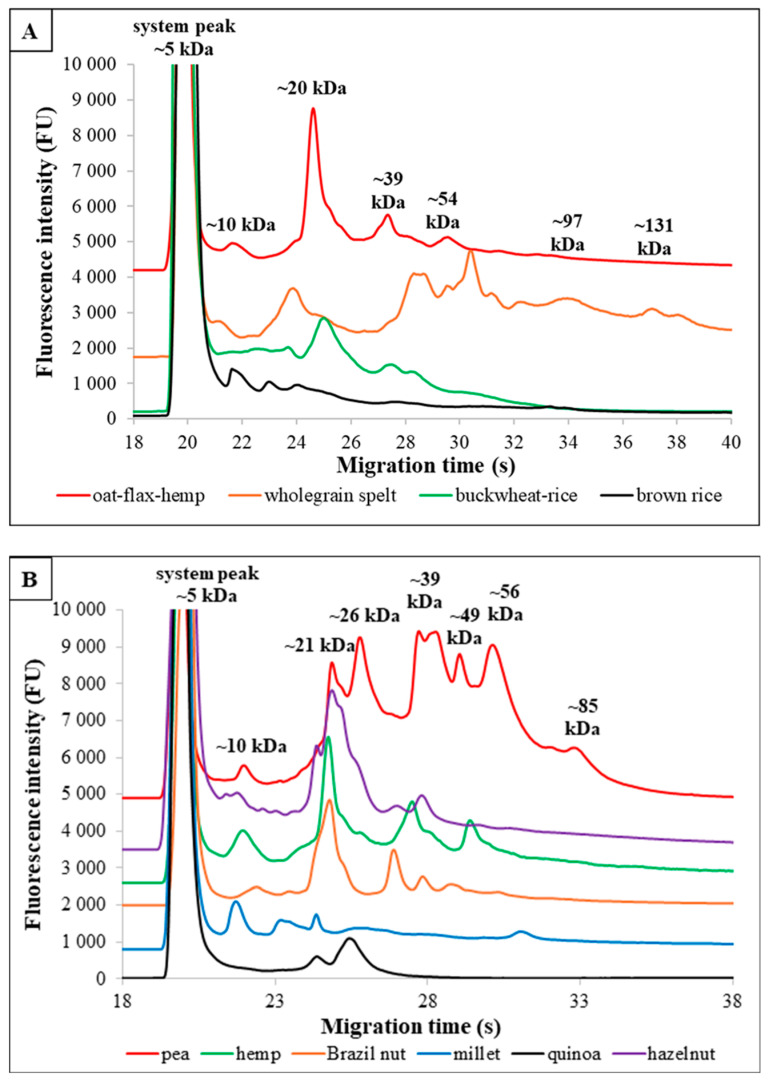
(**A**,**B**) The figures show a comparison of the microfluidic profiles of different plant-based milk replacers. (**A**) illustrates cereal-based, (**B**) additional specific drinks.

The electropherograms of the ten plant-based milk alternatives (Figure 3A,B), which did not have different brands, exhibit unique profiles characterized by distinct patterns of peaks.

Electrophoretic analysis visually represents the variation in TN composition, showcasing the distinct protein profiles of the plant-based beverages. The peaks, representing fractions, vary in number and intensity among the different drinks. This method effectively differentiates between the various plant-based milks based on their TN content. The differences in peak intensities and the number of fractions underscore the heterogeneity among the plant-based milk alternatives.

### 3.5. Statistical Diversity in PBMAs Enriched With Additives

**Figure 4:**
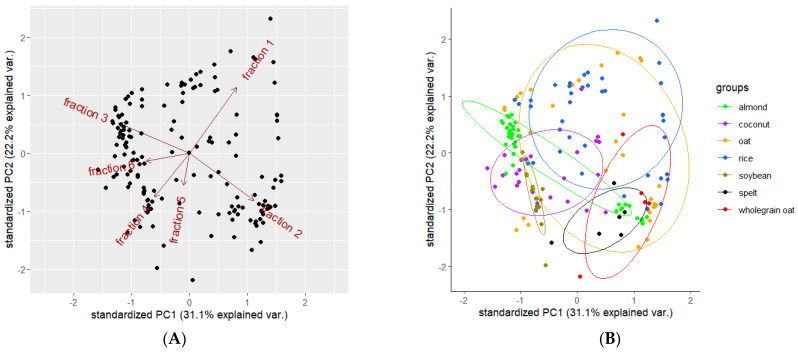
(**A**,**B**) A principal component analysis (PCA) was performed using the percentage of the total time-corrected area of fractions across a range of plant-based beverages. The loading plots (**A**) demonstrate the correlation between principal components PC1 and PC2, indicating the extent to which each variable contributes to these components. The score plots (**B**) represent the percentage of the total time-corrected area of fractions for each plant-based drink, plotted against PC1 and PC2.

Figure 4 illustrates the statistical diversity in the electrophoretic profiles of PBMAs (with at least three different brands of products).

The variability in nitrogen content among different types of plant-based drinks was analyzed using principal component analysis (PCA). The two-dimensional data from the samples were projected onto the plane defined by the two principal components, effectively summarizing the key variations in the dataset. To visualize the statistical significance and dispersion, 95% confidence ellipses were superimposed on the PCA plot, highlighting the regions where the true population mean was likely to lie (Figure 4B). The PCA plot reveals a distinct separation between soybean drink samples (dark green dots) and samples of spelt (black dots), wholegrain oat (red dots), and rice (blue dots). Additionally, there is a distinct separation between spelt drink samples (black dots) and soybean (dark green dots), coconut (purple dots), and rice (blue dots) drink samples. The first and second principal components cumulatively account for 53.3% of the total variability among the products, with PC1 explaining 31.1% and PC2 explaining 22.2%. High inter-product variability was observed for oat (orange dots), rice (blue dots), coconut (purple dots), and wholegrain oat (red dots) drinks (Figure 4B). The first and second fractions of TN content loaded positively on PC1, while fraction 3 showed positive loadings on PC2. Fractions 4, 5 and 6 had negligible effects on both PCs (Figure 4A).

Compared to the results without additives, where the first and second principal components cumulatively account for nearly 62% of the total variability among the products [27], the additive-rich samples showed less clear separation between the samples.

Plant-based drinks show less differentiation and high inter-product variability due to the diverse range of protein and NPN additives used.

## 4. Conclusions

The microchip gel electrophoretic method was found suitable for determining the total nitrogen content of PBMAs containing different additives, with the total nitrogen (TN) value often exceeding the total protein (TP) value stated on the label. Our present study demonstrates that different brands containing various additives, as well as milk alternatives made from specific plants, exhibit unique microfluidic profiles and characteristic features. Furthermore, it was observed that there was a difference in the electrophoretic profiles between additive-free and additive-containing products of PBMAs (oat-based, rice-based, coconut-based, almond-based, soybean-based, and cashew-based drinks) made from the same plant. This difference was reflected in the number and intensity of the components. It is notable that almond milk alternatives exhibited the highest nitrogen concentration and a notable divergence in nitrogen content when compared to the nominal protein content stated on the packaging. This observation can be attributed to the presence of components introduced during the production of almond drinks, which may contribute to the overall nitrogen content.

From a health perspective, the findings of this study provide essential knowledge for patients with mild to advanced kidney disease, for whom reducing the burden on the kidneys is of paramount importance. For individuals with kidney disease, it is of the utmost importance to work closely with healthcare providers, including dietitians, in order to gain a comprehensive understanding of the dietary picture and to make informed choices. While protein intake is an important factor, it is also important to consider the types of additives and their potential contribution to the nitrogen load. It can be reasonably presumed that additives and preservatives in processed foods may indeed contain nitrogen-containing compounds. These compounds can contribute to the overall nitrogen load in the body and, if not adequately excreted by the kidneys, may pose a risk to individuals with kidney disease. Therefore, the total nitrogen content on the food label may be of importance in addition to the protein content. This separation technique represents a novel approach in the fields of analytical chemistry and food chemistry, providing a rapid, low-sample-consumption, reproducible, and adequate method for the determination of total nitrogen content.

## Figures and Tables

**Table 1:** (**A**) Total nitrogen (TN) content in the different plant-based drinks (at least three different brands of products) compared to the nominal total protein concentrations on the packages. Data are expressed as median (interquartile range) values of *n* = 3 products of the same brand, each analyzed in duplicate. Bold *p*-values are statistically significant. Protein and non-protein nitrogen (NPN) additives which can modify the nominal total protein concentration are listed. (**B**) Total nitrogen (TN) content in the different plant-based drinks (there were fewer than three brands available for different drinks) compared to the nominal total protein (TP) concentrations on the packages. Data are expressed as median (interquartile range) values of *n* = 3 products of the same brand, each analyzed in duplicate. Protein and non-protein nitrogen (NPN) additives which can modify the nominal total protein concentration are listed.

(**A**)				
**Product**	**Measured TN** **g/100 mL**	Nominal TP * **g/100 mL**	***p*** **-Value**	**Protein and NPN Additives**
**Oat (*n* = 96)**	2.0 (1.1–6.4)	0.3–1.0	**<0.001**	Sunflower oil, soluble maize grits, chicory root fiber, pea protein, lecithin, safflower oil, B vitamins, sea red algae, marine algae, gellan gum
**Rice (*n* = 120)**	0.7 (0.2–1.2)	0.1–0.5	**<0.001**	Sunflower oil, rapeseed lecithin, gellan gum safflower oil, B vitamins, red algae, marine algae
**Coconut (*n* = 66)**	1.8 (1.2–4.4)	0.0–1.1	**<0.001**	Grape juice concentrate, sunflower lecithin, lecithins, B vitamins, soybean, rice, guargum, gellan gum, xanthan gum
**Soybean (*n* = 42)**	3.7 (1.8–8.3)	2.8–3.4	0.227	Marine algae, red algae, aroma, apple juice, B vitamins, gellan gum
**Almond (*n* = 78)**	8.8 (6.1–12.2)	0.0–0.8	**<0.001**	Sunflower lecithin, carob meal, maize maltodextrin, gellan gum, guargum, maltodextrin, B vitamins, sunflower oil
**Wholegrain oat (*n* = 18)**	8.5 (3.8–9.0)	0.6–0.8	0.080	sunflower oil, marine algae
**Spelt (*n* = 24)**	1.5 (0.7–3.5)	0.7–0.8	0.116	sunflower oil
(**B**)				
**Hazelnut (*n* = 6)**	5.0 (2.4–10.5)	0.4	**0.018**	carob flour, gellan gum, sunflower lecithin, B vitamins
**Cashew (*n* =6)**	5.2 (1.9–14.5)	0.5	**0.026**	carob flour, gellan gum, sunflower lecithin, B vitamins
**Wholegrain spelt (*n* = 6)**	2.8 (2.3–2.9)	0.0	**0.014**	sunflower oil
**Quinoa (*n* = 6)**	1.0 (0.7–1.5)	1.1	0.746	rice, sunflower oil, safflower oil
**Brazil nut (n = 6)**	8.3 (5.1–15.1)	0.7	**0.026**	rice starch, locust bean gum, sunflower lecithin
**Brown rice (*n* = 12)**	0.9 (0.3–2.1)	<0.5	0.122	sunflower oil, safflower oil
**Buckwheat rice (*n* = 6)**	0.8 (0.6–1.4)	0.8	0.916	rice, sunflower oil, safflower oil
**Millet (*n* = 6)**	0.9 (0.8–1.2)	0.7	0.812	sunflower oil
**Hemp (*n* = 6)**	8.6 (6.9–14.2)	0.5	**0.012**	rosemary extract
**Oat–flax–hemp (*n* = 6)**	6.7 (4.3–8.5)	0.7	**0.028**	oat, 4% hemp paste, linseed oil
**Pea (*n* = 6)**	5.5 (2.1–8.1)	1.4	0.113	sunflower oil

* TP: total protein content. Different brands have different total protein concentrations on the label, minimum–maximum values are given.

## Data Availability

The original contributions presented in the study are included in the article, further inquiries can be directed to the corresponding author.
